# Shared Mechanisms Between Osteoarthritis and Cardiovascular Disease: A Clinical and Pathophysiological Review

**DOI:** 10.7759/cureus.86284

**Published:** 2025-06-18

**Authors:** Mohammed Abdul Muqsit Khan, Tabish W Siddiqui, Raqshan W Siddiqui, Syed Muhammad Hayyan Nishat, Asma A Alzaabi, Fatema M Alzaabi, Dana J Al Tarawneh, Yusuf J Al Tarawneh, Abdallah Khan, Shiza W Siddiqui

**Affiliations:** 1 Internal Medicine, Ras Al Khaimah (RAK) Medical and Health Sciences University, Ras Al Khaimah, ARE; 2 Internal Medicine, King Khalid University Hospital, Abha, SAU

**Keywords:** atherosclerosis, cardiovascular diseases, inflammation, obesity, osteoarthritis, oxidative stress

## Abstract

Osteoarthritis (OA) and cardiovascular disease (CVD) are two highly prevalent conditions that often coexist, especially in older adults. This paper explores the complex relationship between OA and CVD, highlighting their shared risk factors, such as aging, obesity, inflammation, and metabolic dysfunction. It delves into how age-related changes in both the joints and the cardiovascular system contribute to disease progression. The role of oxidative stress, chondrocyte senescence, and endothelial dysfunction are examined as key mechanisms linking these conditions. Pharmacological treatments commonly used in OA, including non-steroidal anti-inflammatory drugs (NSAIDs) and corticosteroids, are discussed in terms of their cardiovascular implications. In addition, the paper reviews current diagnostic approaches for CVD in OA patients and suggests that a more comprehensive and personalized strategy is needed. It also evaluates lifestyle and pharmacologic interventions that could benefit both joint and cardiovascular health. While evidence supports a connection between OA and increased cardiovascular risk, the paper emphasizes the need for further research to clarify causation, improve risk prediction, and guide multidisciplinary treatment strategies. Ultimately, integrating cardiovascular care into OA management can lead to better outcomes and quality of life for affected individuals.

## Introduction and background

As of 2020, osteoarthritis (OA) affects 7.6% of the global population, with a higher incidence in females (8,058.9 per 100,000) than males (5,780.1 per 100,000), and increasing prevalence with age [1,2]. Over the past 30 years, OA cases have surged by 132.2%, with projections indicating a further 60-100% rise in the next three decades [2]. Cardiovascular diseases (CVDs) continue to be a major global health burden, causing approximately 17.9 million deaths annually [3]. These include ischemic heart disease, heart failure, arrhythmias, valvular diseases, and stroke [3], and together account for nearly one-third of all global deaths [4]. Ischemic heart disease remains the leading cause of mortality among non-communicable diseases, responsible for 9.14 million deaths worldwide [5].

Both modifiable and non-modifiable risk factors influence CVD development [6]. Key behavioral risk factors include an unhealthy diet, physical inactivity, and smoking. These, in turn, contribute to elevated blood pressure, glucose, and lipid levels, further increasing cardiovascular risk [6].

Although OA is primarily recognized as a musculoskeletal disorder, emerging evidence suggests a significant impact on cardiovascular health [7]. Individuals with OA, particularly those with knee and hip involvement, exhibit a higher prevalence of CVDs [7]. The association between OA and CVD appears to be stronger in women than in men [8]. Furthermore, OA has been linked to an increased risk of heart failure, new-onset coronary artery disease, and CVD-related hospitalizations [8].

This review aims to explore the mechanisms through which OA contributes to cardiovascular impairment and modifies established risk factors. Additionally, it will provide a brief overview of diagnostic approaches and management strategies.

## Review

Methodology

This review was conducted in accordance with the Preferred Reporting Items for Systematic Reviews and Meta-Analyses (PRISMA) guidelines. A comprehensive search was carried out across PubMed, Scopus, and Google Scholar to identify relevant studies examining the association between osteoarthritis (OA) and cardiovascular diseases (CVDs). The search strategy incorporated keywords such as "osteoarthritis and cardiovascular disease," "OA and metabolic syndrome," and "NSAIDs and CVD risk," using Boolean operators (AND, OR) to optimize results. The search was limited to articles published between January 2000 and December 2024 to ensure contemporary relevance.

A total of 200 records were retrieved from the databases: 140 from PubMed, 40 from Scopus, and 20 from Google Scholar. After removing 20 duplicate records, 180 studies underwent initial screening. Based on predefined inclusion and exclusion criteria (mentioned below), 140 studies were excluded due to irrelevance, being case reports or reviews, or lacking peer review.

**Figure 1:**
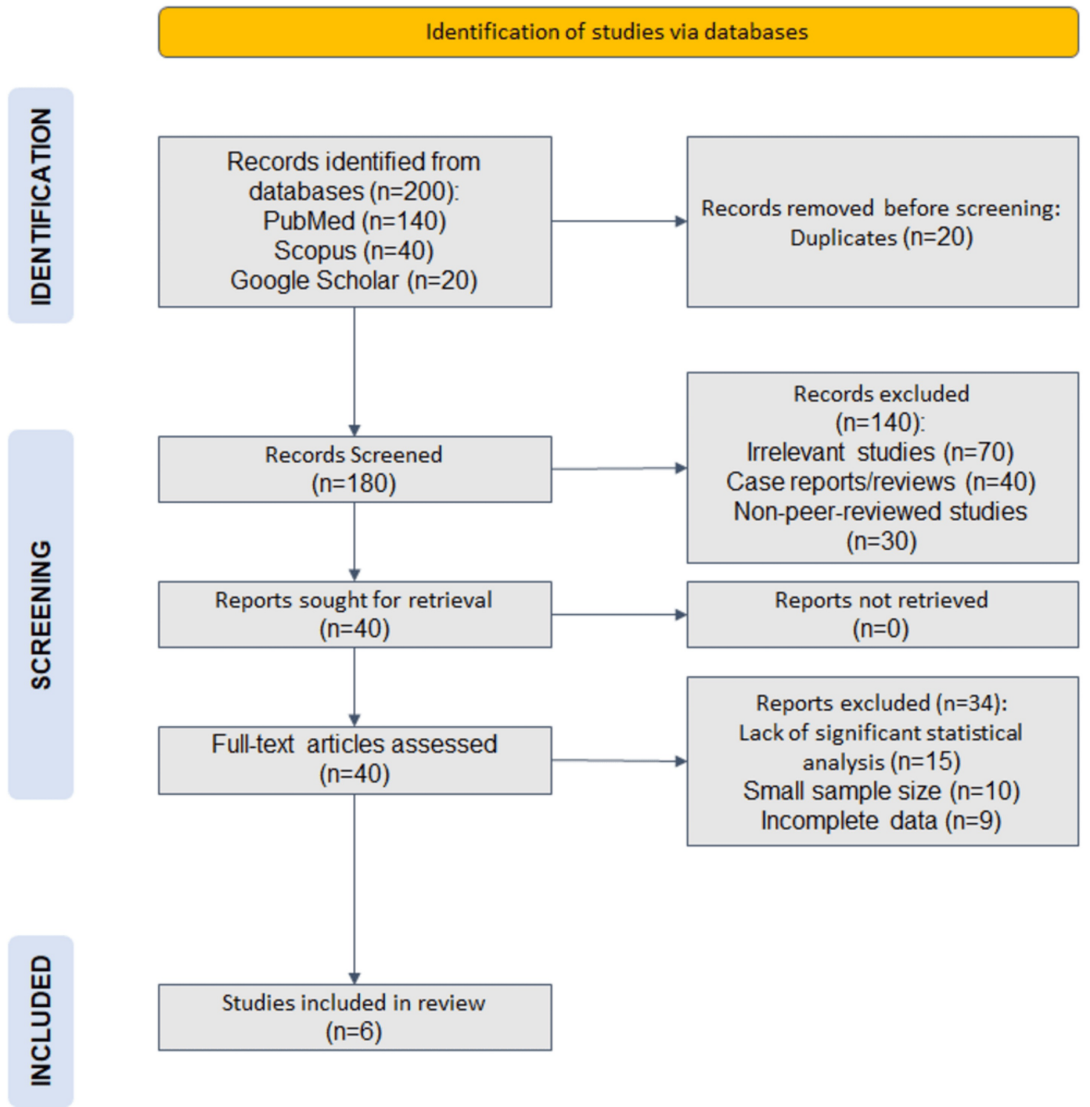
PRISMA flowchart of selected articles. PRISMA: Preferred Reporting Items for Systematic Reviews and Meta-Analyses.

The remaining 40 full-text articles were assessed for eligibility, of which 34 were excluded due to lack of significant statistical analysis (n=15), small sample sizes (n=10), or incomplete data (n=9). As a result, six studies met the inclusion criteria and were selected for the final review. Although only a small number of studies were included from a large initial pool, this was due to the application of strict eligibility criteria to ensure methodological rigor and relevance. The review focused exclusively on high-quality observational studies with primary data, robust statistical analysis, and a clear investigation of the OA-CVD relationship, aiming to synthesize the most reliable evidence available. The PRISMA flow diagram illustrates this selection process. PRISMA chart has been demonstrated in Figure 1.

Inclusion and Exclusion Criteria

The inclusion criteria comprised observational studies--specifically cross-sectional, cohort, case-control designs, or Mendelian studies--that investigated the association between osteoarthritis and cardiovascular disease. Only studies published between January 2000 and December 2024 were considered, and those involving an adult population aged 18 years or older were included to maintain demographic consistency. Eligible studies explicitly examined shared pathophysiological mechanisms, such as inflammation or metabolic syndrome, or assessed the impact of pharmacological interventions, including non-steroidal anti-inflammatory drug (NSAID) use. To ensure methodological rigor, only peer-reviewed articles published in English that presented primary data and employed robust statistical analysis were selected.

The exclusion criteria followed PRISMA recommendations, omitting studies that did not directly examine the OA-CVD connection (n=70), including those unrelated to metabolic syndrome, inflammatory pathways, or pharmacological treatments. Case reports, narrative reviews, and systematic reviews without original data were excluded (n=40), as were non-peer-reviewed studies, such as conference abstracts or editorials (n=30). Furthermore, studies lacking significant statistical analysis (n=15) or those with small sample sizes that limited generalizability (n=10) were removed. Lastly, studies with incomplete or missing data that affected validity and reproducibility (n=9) were excluded. After applying these inclusion and exclusion criteria, six studies were selected for the final review.

Risk of Bias Assessment

The quality of the included observational studies was assessed using the Newcastle-Ottawa Scale (NOS), a validated tool for evaluating non-randomized studies. The NOS assessed three key domains: selection of study groups, comparability of groups, and assessment of outcome or exposure. Each study was scored out of a maximum of nine points. To ensure methodological rigor, only studies scoring six or higher--indicating moderate to high quality--were included in the final review. Particular attention was given to whether studies adequately controlled for major confounders such as age, sex, and comorbidities, which are especially relevant in OA and CVD research.

Pathophysiology

OA is classified into two primary types: primary and secondary [1]. Primary OA is diagnosed in the absence of any predisposing joint trauma or disease, although patients may present with risk factors such as high body mass, repetitive joint stress due to occupational activities, muscle weakness, or metabolic syndrome [9]. Secondary OA, on the other hand, occurs in the presence of pre-existing joint pathology, including prior joint injuries, inflammatory arthritis, septic arthritis, congenital disorders such as Ehlers-Danlos or Marfan syndrome, metabolic syndromes, or age-related conditions like osteoporosis [9].

Regardless of its etiology, OA initially affects the articular cartilage, leading to surface fibrillation and progressive erosion [9]. As degeneration advances, the structural integrity of the collagen matrix becomes compromised, triggering chondrocyte proliferation [9]. Disease progression eventually results in cartilage ossification and osteophyte formation [9]. Simultaneously, subchondral bone undergoes thickening due to increased collagen mineralization [9], and in rare cases, bony cysts and erosions may develop [9].

**Figure 2:**
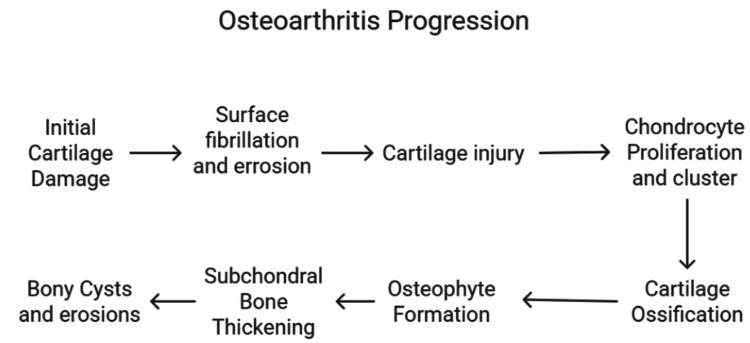
Describing OA pathophysiology. Illustrated by Mohammed Abdul Muqsit Khan. OA: osteoarthritis.

Although OA and CVD have traditionally been regarded as distinct conditions, a significant proportion of individuals experience both concurrently [10]. The link between these diseases can be understood by examining shared risk factors, common etiological mechanisms, and symptom interactions, whereby OA may contribute to the worsening of CVD [10]. The pathophysiology of OA has been illustrated in Figure 2.

Common Pathophysiological and Risk Factors Linking Osteoarthritis and Cardiovascular Disease

OA and CVD share common risk factors, including atherosclerosis (ATH), metabolic syndrome, chronic inflammation, obesity, and advanced glycation end products (AGEs) [10]. These factors contribute to both joint degeneration and cardiovascular damage, highlighting the need for integrated management strategies [10].

Atherosclerosis: A Shared Mechanism Linking Osteoarthritis and Cardiovascular Disease

ATH represents a critical link in the shared pathophysiological mechanisms underlying OA and CVDs [8]. Studies have demonstrated an independent correlation between OA in the distal interphalangeal (DIP), metacarpophalangeal (MCP), and knee joints and the presence of ATH, as evidenced by carotid plaque formation or intima-media thickening in women; however, this association has not been observed in men [8]. In a study conducted by Jonsson et al., a significant correlation was identified between hand osteoarthritis and atherosclerosis in older women [11]. The authors postulated that circulatory abnormalities affecting the synovial membrane and subchondral bone may contribute to the pathophysiological progression of OA and cartilage degradation [11].

A strong association also exists between obesity and both ATH and OA [12]. While OA in weight-bearing joints is largely attributed to mechanical stress resulting from excess body weight, the occurrence of OA in non-weight-bearing joints (e.g., the hands) suggests a metabolic component in the form of pro-inflammatory mediators [12]. This implies that systemic inflammatory and metabolic pathways triggered by obesity play a significant role in the development of both OA and ATH [12].

Excess lipids and cholesterol exacerbate inflammation, a central mechanism in the pathogenesis of both OA and ATH [13]. Dyslipidemia, defined by abnormal lipid profiles, is recognized as a major risk factor for both conditions [13]. In particular, hypercholesterolemia has been implicated in the development of generalized OA by promoting oxidative stress, inflammation, and lipid accumulation in joint tissues, which in turn induces chondrocyte apoptosis and cartilage degradation [13]. To mitigate potential cytotoxicity, lipid metabolism--especially the regulation of free cholesterol--is tightly regulated at the cellular level [13]. Supporting this, lipid deposits have been observed in osteoarthritic cartilage, and fatty acids are known to contribute to OA progression [13]. Adipokines, such as adiponectin, are secreted in response to obesity and play a significant role in metabolic regulation and inflammatory pathways [13]. These molecules are implicated in both vascular remodeling in ATH and joint degeneration in OA, reinforcing the shared inflammatory basis of the two diseases [13]. Recognizing OA as a disease associated with metabolic syndrome further highlights its systemic nature, extending beyond mechanical wear and tear [13].

Moreover, elevated levels of endothelin-1 (ET-1), a potent vasoconstrictor, have been linked to ATH and are believed to contribute to cartilage degradation by stimulating matrix-degrading enzymes [14]. ET-1 also induces the release of vascular endothelial growth factor (VEGF), which serves as an early biomarker of cartilage and subchondral bone damage [14]. VEGF, in turn, facilitates atherosclerotic plaque development, underscoring a potential mechanistic overlap between OA and CVD [14].

Metabolic Syndrome and Its Role in the Development of Osteoarthritis: Insights into a Complex Relationship

Metabolic syndrome comprises five key components: hypertension, abdominal obesity, hypertriglyceridemia, hyperglycemia, and low levels of high-density lipoprotein (HDL) [15]. A diagnosis of metabolic syndrome is established when an individual presents with three or more of these risk factors [16]. However, even the presence of a single component significantly increases the risk of CVD [16]. Individuals diagnosed with metabolic syndrome face a substantially higher risk of developing major cardiovascular events, including myocardial infarction (MI) and stroke [16].

Data from the National Health and Nutrition Examination Survey (NHANES) III indicate that 59% of individuals with OA also had metabolic syndrome, compared to 29% of individuals without OA [17]. The association between OA and metabolic risk factors appears particularly pronounced in younger individuals, with OA presence by the age of 43.8 years linked to a 5.26-fold increased risk of developing metabolic syndrome [17]. Moreover, recent studies indicate that the incidence and severity of knee and hand OA are significantly higher in obese individuals with metabolic syndrome, while diabetes mellitus (DM) and hypertension (HTN) have also been consistently associated with an independent increase in the incidence, prevalence, and severity of OA [18-21].

**Figure 3:**
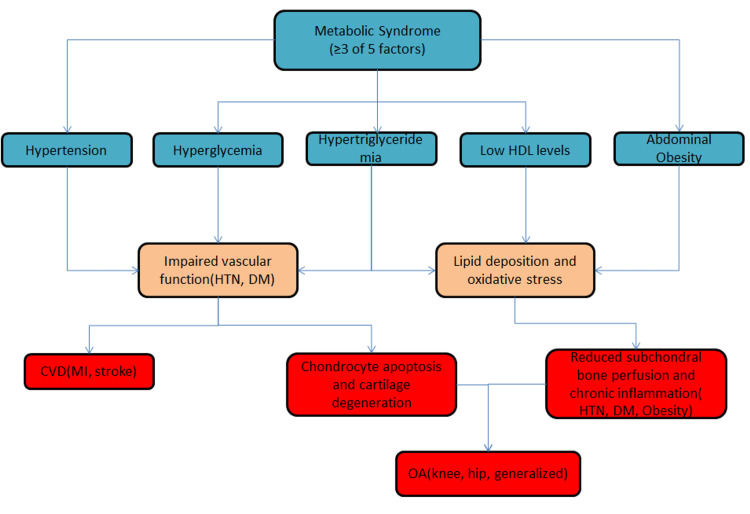
Association of metabolic syndrome with OA and CVD. HDL: high-density lipoproteins; HTN: hypertension; DM: diabetes mellitus; CVD: cardiovascular diseases; MI: myocardial infarction; OA: osteoarthritis; describing OA and metabolic syndrome relation. Illustrated by Mohammed Abdul Muqsit Khan.

HTN and DM accelerate both CVD and OA by impairing vascular function and microcirculation, reducing subchondral bone perfusion, and promoting chronic inflammation, which exacerbates cartilage degeneration and cardiovascular complications [22-24]. The association of metabolic syndrome with OA and CVD has been demonstrated in Figure 3.

The Interplay of Osteoarthritis, Obesity, and Cardiovascular Disease

OA and CVD share complex interconnections, with obesity acting as a key modifiable risk factor that exacerbates both conditions [3]. The predominant symptoms of OA include pain, stiffness, and restricted joint motion, which initially improve with rest but worsen as joint degeneration progresses [25,26]. Structural damage in weight-bearing joints, such as the knees and hips, leads to persistent discomfort, swelling, and a decline in functional mobility, further weakening surrounding musculature and exacerbating joint stress [1,26]. Although structured physical exercise regimens can alleviate pain, enhance joint function, and delay disability progression, adherence is often hindered by pain-related limitations [26,27].

A sedentary lifestyle, frequently observed in individuals with OA, significantly increases the risk of CVD [28]. Physical inactivity is a major contributor to cardiovascular complications, whereas even low levels of structured movement, such as walking programs, have been shown to mitigate CVD risk [29]. Meta-analyses reveal strong associations between OA, diabetes mellitus (DM), and CVD, with OA patients exhibiting a higher incidence of DM, ischemic heart disease (IHD), and heart failure (HF) [30].

Obesity remains the most significant modifiable risk factor for OA, with individuals having a body mass index (BMI) >30 kg/m² being 6.8 times more likely to develop OA compared to those with a normal BMI [31]. Mechanically, excess weight accelerates joint degeneration by increasing stress on weight-bearing joints while reducing muscle strength and hastening structural damage [31]. However, obesity's role in OA extends beyond mechanical loading, as systemic metabolic alterations and inflammation contribute to disease progression [12]. This is further evidenced by the twofold increased risk of OA in non-weight-bearing joints, such as the hands, among obese individuals [12].

Adipose tissue plays an active role in the pathophysiology of both OA and CVD by releasing adipokines, such as leptin, adiponectin, and resistin, which disrupt cartilage homeostasis and promote joint degradation [22,32,33]. In obesity, an imbalance in adipokine production results in elevated pro-inflammatory mediators (e.g., tumor necrosis factor alpha (TNF-α) and interleukin-6 (IL-6)) and decreased cardioprotective adipokines, such as adiponectin, fostering systemic inflammation and apoptosis [33].

Interleukin-6 (IL-6) and C-reactive protein (CRP) are key inflammatory biomarkers that underscore the metabolic-inflammatory link between OA and CVD [32,33]. IL-6, a pro-inflammatory cytokine, is elevated in OA--particularly among obese individuals--and correlates with cartilage degradation markers like Helix-II and worsening Western Ontario and McMaster Universities Osteoarthritis Index (WOMAC) scores [33]. It reflects both local joint inflammation and systemic metabolic dysregulation [33]. CRP, a downstream acute-phase reactant, is similarly elevated in OA and is associated with endothelial dysfunction and atherosclerosis progression, reinforcing its relevance to cardiovascular risk [32,33]. Both biomarkers respond to weight loss interventions, with associated improvements in cartilage metabolism markers [32,33]. Together, IL-6 and CRP serve as valuable indicators of inflammatory and metabolic activity in OA with cardiovascular implications [32,33].

**Figure 4:**
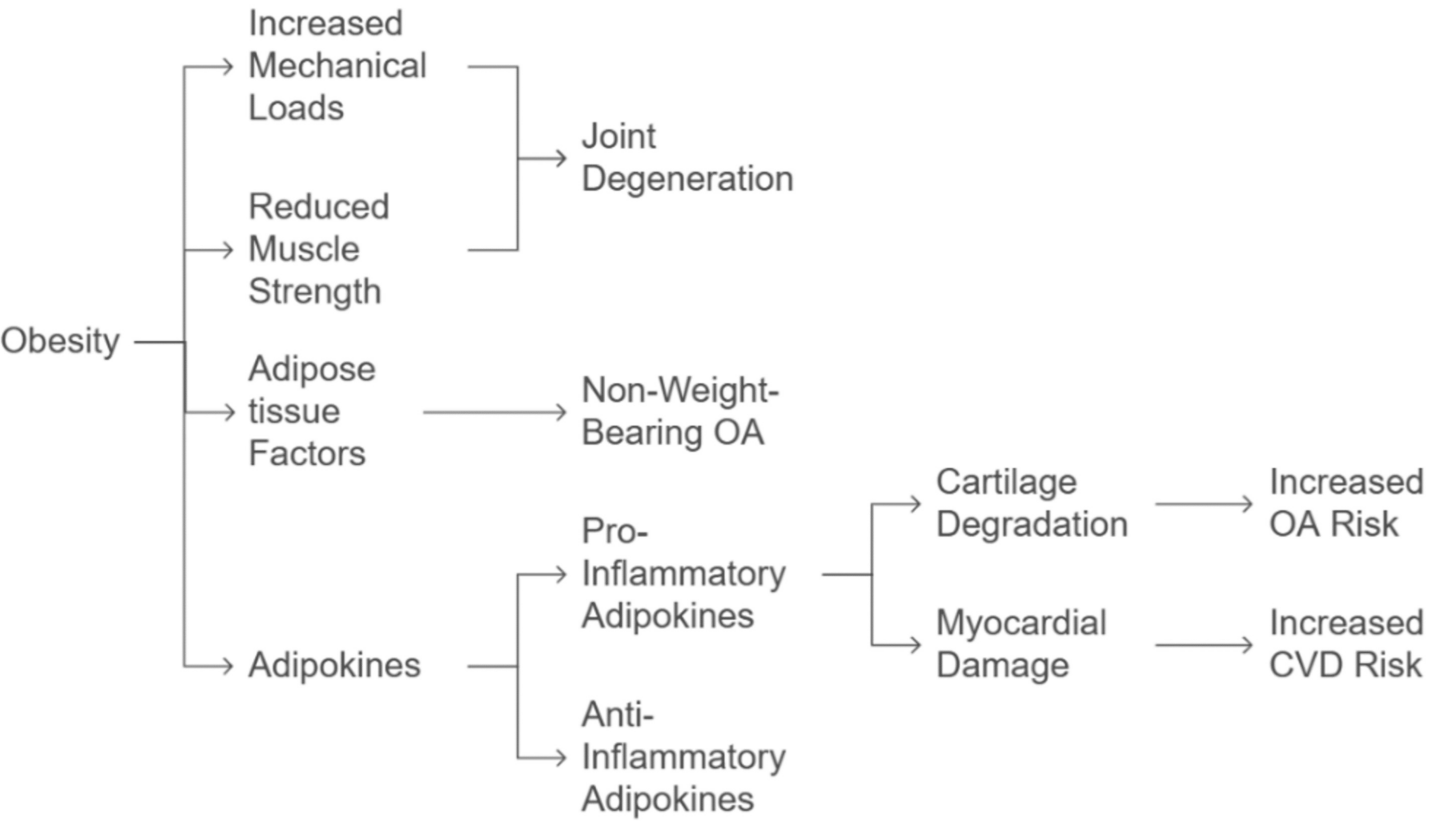
Role of obesity in OA and CVD. OA: osteoarthritis; CVD: cardiovascular disease. Illustrated by: Mohammed Abdul Muqsit Khan.

Given these interconnected mechanisms, targeting obesity and promoting physical activity are essential strategies for mitigating both OA and CVD. Comprehensive lifestyle interventions focusing on weight management, structured exercise, and inflammation control may help break this detrimental cycle, improving overall musculoskeletal and cardiovascular health. The role of obesity in OA and CVD has been illustrated in Figure 4.

Chronic Inflammation: The Silent Bridge Connecting Osteoarthritis and Systemic Diseases

Chronic inflammation serves as a key underlying factor linking OA and CVD, as it significantly contributes to the pathophysiology of both conditions [22].

In OA, chronic inflammation is driven by the local production of inflammatory mediators, including cytokines such as interleukin-1β (IL-1β) [23]. These mediators contribute to cartilage degradation and synovial activation, leading to progressive joint damage [23]. Furthermore, inflammation within joint tissues can exert systemic effects, as evidenced by elevated levels of inflammatory markers in plasma and peripheral blood leukocytes (PBLs) of OA patients [23]. Gene expression profiling of PBLs in OA has identified subsets with an "IL-1β signature," which is associated with increased pain severity and a heightened risk of disease progression [24].

Similarly, CVD, particularly ATH, is now recognized as a chronic inflammatory disorder [25]. The accumulation of low-density lipoprotein (LDL) cholesterol in arterial walls provokes an inflammatory response, perpetuating vascular dysfunction [25]. Even when LDL cholesterol levels are effectively controlled, residual risk for major adverse cardiovascular events (MACE) persists, primarily due to ongoing inflammation [25]. Clinical trials, such as the Canakinumab Anti-inflammatory Thrombosis Outcome Study (CANTOS) trial, have demonstrated that targeting IL-1β with canakinumab significantly reduces MACE, further emphasizing the critical role of inflammation in CVD pathogenesis [25].

Another key factor linking OA and CVD is the accumulation of advanced glycation end products (AGEs) [10]. AGEs progressively accumulate in cartilage and vascular tissues, inducing collagen cross-linking [10]. In OA, this process contributes to joint stiffening and chronic inflammation, while in CVD, systemic AGE accumulation leads to arterial stiffening, increasing the risk of hypertension--a major predisposing factor for CVD [10]. Notably, epidemiological studies have reported elevated hypertension prevalence in individuals with OA, reinforcing the interconnection between these diseases [10].

Thus, chronic inflammation serves as a unifying mechanism underlying both OA and CVD, with systemic inflammatory responses, cytokine activity, and AGE accumulation contributing to their pathophysiology.

The Role of Aging in Osteoarthritis and Cardiovascular Disease

OA, a degenerative joint disease primarily affecting older adults, shares common risk factors with CVD, particularly the physiological changes associated with aging. Age is a key determinant of cardiovascular health, and as individuals grow older, the heart and vascular system undergo structural and functional changes that elevate the risk of conditions such as ATH, HT, MI, and stroke [13]. Aging-related changes in cardiovascular tissues include hypertrophy, altered left ventricular (LV) diastolic function, decreased LV systolic reserve, arterial stiffness, and reduced endothelial function [13]. Furthermore, aging leads to a reduction in the number of cardiomyocytes due to increased apoptosis and necrosis, as well as a decreased ability to regenerate cardiomyocytes from cardiac stem cells [13]. These changes profoundly affect the cellular composition of the heart [13]. Cardiomyocytes become more susceptible to stress, especially oxidative stress, as they age [13]. This results in an increase in ROS production, leading to heightened oxidative stress and a higher rate of cardiomyocyte death [13]. The release of cellular debris from dying cardiomyocytes can negatively impact neighboring cells and foster a proinflammatory, profibrotic environment in the aging heart [13].

Inflammation in OA is driven by both external factors, such as cytokines and proteases, and internal processes that result in the accumulation of oxidized proteins [34,35]. These oxidized proteins increase the production of reactive oxygen species (ROS), which cause oxidative damage and fuel further inflammatory responses [35]. This oxidative stress accelerates the process of chondrocyte senescence, leading to a loss of their functional abilities [35]. OA is mainly considered an age-related disorder, and as chondrocytes age, they display the senescence-associated secretory phenotype (SASP), a phenotype triggered by inflammatory mediators from oxidative stress [35]. This leads to the increased release of inflammatory cytokines and enzymes like MMP-13 [35]. Additionally, AGEs, which accumulate in aging tissues, interact with receptors for AGE (RAGE) on chondrocytes, causing the release of more inflammatory molecules and further contributing to cartilage degradation [35].

The Hormonal Axis in Osteoarthritis and Cardiovascular Disease

Sex hormones, particularly estrogen and testosterone, play a critical role in modulating the risk and pathophysiology of both OA and CVD [7,8,10]. Premenopausal women, with higher circulating estrogen levels, tend to have more favorable fat distribution profiles--predominantly subcutaneous rather than visceral--and lower CVD risk compared to men. This hormonal protection appears to diminish after menopause, as estrogen levels decline, leading to increased visceral adiposity, elevated blood pressure, lipid dysregulation, and higher susceptibility to both CVD and OA [7,10].

A parallel rise in OA prevalence in postmenopausal women further supports the musculoskeletal protective effect of estrogen. Studies have shown a significant association between OA and atherosclerosis in women, particularly at menopausal transition when hormonal shifts are most pronounced [7,10]. Moreover, the decline in estrogen correlates with increased pain sensitivity, possibly explaining the higher pain burden and OA symptom reporting in women compared to men in the 40-60 age group [8,10].

Testosterone receptors are present in chondrocytes of both sexes, but testosterone appears to exert cartilage-modulatory effects primarily in men, suggesting sex-specific hormonal interactions in joint health [8,10]. While estrogen’s precise role in nociceptive modulation remains debated, evidence points to hormonal fluctuations influencing both structural joint changes and pain perception [8,10].

These hormonal dynamics underscore a common etiological link between OA and CVD in women, particularly after menopause, and highlight the need for sex-specific prevention and treatment strategies targeting shared metabolic and inflammatory pathways [10].

Pharmacological Interventions and Their Cardiovascular Implications

Non-steroidal anti-inflammatory drugs (NSAIDs) and cyclo-oxygenase-2 (COX-2) inhibitors are among the most commonly prescribed medications for the management of OA to alleviate pain [36]. NSAIDs exert their effects by inhibiting the production of prostaglandins and thromboxane A; while corticosteroids do so by inhibition of cyclo-oxygenase at the level of mRNA [36]. Notably, approximately 40% of OA patients also have a concurrent diagnosis of hypertension [37]. The use of NSAIDs and COX-2 inhibitors may result in an increase in blood pressure (BP), a response that is particularly pronounced in individuals with pre-existing HT [37]. Furthermore, many antihypertensive medications exhibit reduced efficacy when used concomitantly with NSAIDs and COX-2 inhibitors [37]. The inhibition of cyclo-oxygenase (COX) by these medications reduces the vasodilatory effects of prostaglandins PGE2 and PGI2, which may contribute to vasoconstriction and strong anti-natriuretic effects [37]. These mechanisms significantly elevate the risk of hypertension [37]. Additionally, renal toxicity is another adverse effect that exacerbates stress on the cardiovascular system by activating the renin-angiotensin-aldosterone system (RAAS) [37].

Intra-articular corticosteroids are widely used for pain relief in symptomatic OA [38]. Their effects typically last for two to four weeks, necessitating repeated injections, often up to four times per year [38]. It is well-recognized that corticosteroids have the potential to exacerbate coagulopathy, hyperlipidemia, HT, and hyperglycemia [38]. Additionally, the presence of glucocorticoid receptors in cardiovascular tissues increases the likelihood of localized effects on ATH [38]. Blood pressure and blood glucose levels tend to rise in the hours and days following injections [38]. There have also been reports of decreased blood salicylate levels shortly after corticosteroid injections, which may have important implications for patients with acute coronary syndrome (ACS) [39].

Demographic and clinical profile of osteoarthritis (OA)-cardiovascular disease (CVD)

The demographic and clinical profile of the OA-CVD association has been demonstrated in Table 1.

**Table 1:** Demographic table demonstrating the link between OA and CVD. OA: osteoarthritis; CVD: cardiovascular disease; CHD: congenital heart disease; HF: heart failure; USA: United States of America.

Study	Study design	Sample size	Geography	OA type	CVD type	Main findings	Conclusion
Uematsu et al., 2024 [40]	Population-based matched case-control study	~1 million patients	Japan	Hip OA, knee OA, and hand OA	Ischemic heart disease, stroke, congestive heart failure	Cardiovascular risk appears to be highest in patients with knee OA, followed by hip and hand OA, indicating a potential site-specific influence on disease burden.	Knee OA was associated with an increased risk of CVD, particularly for ischemic heart disease and stroke.
Wang et al., 2022 [41]	Mendelian randomization	796,997 OA patients, 103,759 CVD patients	Global (Europe an ancestry)	Hip and knee OA	CHD, HF, and stroke	The risk of heart failure was modestly increased, with a slightly higher association observed for stroke.	Hip OA increased the incidence of HF and stroke significantly. CHD also has a causal relationship with the risk of knee OA.
Hall et al. (2015) [42]	Systematic review and meta-analysis	32,278,744 individuals	Global	General OA	HF, ischemic heart disease, myocardial infarction, stroke.	OA patients had a higher risk of heart failure and ischemic heart disease, but no increased risk of myocardial infarction or stroke. A decreased risk of transient ischemic attack was noted.	Significant prevalence of CVD in OA patients, but the complex relationship suggests shared underlying mechanisms needing further study.
Hoeven et al. (2014) [43]	Prospective cohort study	4648 participants	Netherlands	Radiographic and clinical OA	Coronary heart disease and stroke	Radiographic OA was not linked to CVD risk, and clinical OA showed no significant risk increase. However, disability correlated with higher CVD risk, independent of OA presence.	OA itself was not a risk factor for CVD, but disability linked to OA might influence cardiovascular outcomes, suggesting a need for further research.
Rahman et al. (2013) [22]	Cross-sectional study	40,817 OA patients	USA	Hip and knee OA	Ischemic heart disease, heart failure, stroke	OA patients had a significantly higher risk of developing heart disease, stroke, and heart failure compared to non-OA patients.	The study emphasizes the heightened risk of cardiovascular conditions among OA patients, suggesting shared pathophysiological mechanisms and the need for integrated care.
Ravi et al. (2013) [44]	Propensity score matched landmark analysis	2200 participants	Canada	Hip OA and knee OA	Serious cardiovascular events	Patients who underwent total joint arthroplasty had a 44% lower risk of cardiovascular events compared to those who did not, with an absolute risk reduction of 12.4% over seven years.	Total joint arthroplasty in moderate-severe OA patients may offer significant cardioprotective benefits, suggesting it as a viable intervention for reducing CVD risk.

The population table encompasses a diverse array of studies investigating the link between OA and CVD, providing insight into a complex and multidimensional relationship. The studies vary significantly in design, ranging from population-based case-control and longitudinal cohort studies to Mendelian randomization and large-scale meta-analyses, collectively covering millions of individuals across different regions and OA subtypes.

Uematsu et al. conducted a population-based matched case-control study in Japan, analyzing approximately one million patients to investigate the association between OA and CVD. The study focused on hip, knee, and hand OA and examined their relationship with ischemic heart disease, stroke, and congestive heart failure [40]. The findings indicated increased odds for CVD in OA patients, with knee OA showing the highest association, followed by hip OA and hand OA [40]. Notably, knee OA was particularly linked to ischemic heart disease and stroke, leading to the conclusion that knee OA significantly elevates the risk of CVD [40].

Wang et al. utilized Mendelian randomization in a global study focusing on individuals of European ancestry, specifically evaluating hip and knee OA in relation to coronary heart disease (CHD), heart failure (HF), and stroke [41]. The study found that hip OA was associated with an increased risk of HF and stroke, while CHD exhibited a causal link with knee OA [41].

Rahman et al. conducted a cross-sectional study in the USA, examining the association between hip and knee OA and various cardiovascular conditions, including ischemic heart disease, HF, and stroke [22]. The results showed that OA patients had significantly higher odds of developing heart disease, stroke, and HF compared to individuals without OA [22].

Even though the studies conducted by Uematsu et al., Wang et al., and Rahman et al. employed different methodologies, study designs, and classification criteria for OA and CVD, they all consistently suggest a link between OA and CVD [40]. Knee and hip OA appear most strongly associated with cardiovascular events, particularly ischemic heart disease and stroke [22,40,41].

These findings align with those of Hall et al., a large-scale systematic review and meta-analysis, which reported an elevated risk of HF and ischemic heart disease in OA patients but no increased risk of myocardial infarction or stroke [42].

In contrast, Hoeven et al., in a prospective cohort study, found that radiographic OA classified using the Kellgren-Lawrence scale was not significantly linked to increased CVD risk, and clinical OA only had a minimal risk elevation [43]. However, the study highlighted that disability associated with OA was a significant predictor of cardiovascular events [43]. This suggests that while OA itself may not always be a direct causative factor, the associated functional impairment, reduced mobility, and systemic inflammation could contribute to worsening cardiovascular health [43].

Ravi et al. indicate that total joint arthroplasty (TJA) may offer long-term cardioprotective benefits in patients with advanced osteoarthritis [44]. Despite the need for careful management of short-term postoperative risks, TJA appears to be associated with a reduced incidence of cardiovascular events over time. These findings support its potential role beyond symptom relief, particularly in OA patients with elevated cardiovascular risk [44].

Optimizing CVD diagnosis in OA patients: Methods and insights 

Patients with OA frequently undergo cardiovascular assessments that include both standard evaluations and specialized tests tailored to the complexities of OA [45]. Standard diagnostic procedures include a comprehensive lipid profile-measuring total cholesterol, high-density lipoprotein (HDL), low-density lipoprotein (LDL), and triglycerides-as well as blood pressure monitoring to detect HT, a major risk factor for CVD [45]. Additionally, blood glucose testing aids in the diagnosis of diabetes, a condition known to be associated with increased cardiovascular risk [40,45,46].

The Framingham Risk Score is a widely utilized tool for estimating a patient's 10-year CVD risk, incorporating factors such as age, sex, blood pressure, cholesterol levels, smoking status, and diabetes [46]. This scoring system is instrumental in guiding preventive strategies and treatment decisions and has been applied to assess CVD risk in OA patients [46].

Advanced cardiovascular assessments may include an echocardiogram to evaluate cardiac function and detect structural abnormalities, as well as an electrocardiogram (ECG/EKG) to assess the electrical activity of the heart and identify potential arrhythmias [45,47]. Additionally, physical function tests, such as the "get up and go" test or stair-climb assessments, may be employed in OA patients to evaluate functional impairments that could contribute to an elevated cardiovascular risk [45,46]. In patients with severe symptomatic OA, cardiopulmonary stress testing using arm pedals may be utilized to assess cardiorespiratory fitness [48]. Collectively, these diagnostic modalities provide a comprehensive evaluation, facilitating the effective management of CVD risk in individuals with OA [45].

Improving cardiovascular health in osteoarthritis patients

Physical activity, including closed and open kinetic chain exercises as well as aerobic training in semi-submerged water environments, has been shown to significantly reduce pain, enhance muscle strength, and improve WOMAC scores in OA patients, regardless of CVD risk status [46]. Aquatic therapy, in particular, offers joint-friendly resistance and cardiovascular stimulation, while aerobic exercises, such as walking or swimming, further support metabolic and cardiovascular health [46]. Effective cardiovascular management in OA requires a comprehensive approach that extends beyond traditional physical activity and weight control, incorporating structured interventions such as aquatic therapy and aerobic exercise to optimize both joint function and heart health [46]. The following additional strategies are crucial for further improving cardiovascular outcomes in OA patients.

Comprehensive Risk Assessment in Osteoarthritis for Cardiovascular Disease Prevention

A multidisciplinary approach is essential for optimizing cardiovascular health in patients with severe OA. Lifestyle modifications play a critical role in improving cardiovascular outcomes, including dietary adjustments, engagement in regular low-impact exercise, and smoking cessation [46]. Pharmacological management often involves medications to regulate blood glucose, blood pressure, and cholesterol levels; however, these pharmacological interventions are carefully selected to avoid agents that may exacerbate OA symptoms [40,45]. Weight management is a fundamental component of OA treatment, as reducing excess body weight alleviates joint loading, enhances mobility, and decreases systemic inflammation, ultimately lowering cardiovascular risk [46]. In severe cases, surgical interventions, such as total joint arthroplasty, may indirectly contribute to improved cardiovascular health by restoring mobility and enhancing overall physical function [45].

A comprehensive assessment of cardiovascular risk factors should be a routine component of every clinical examination for OA patients, given the high prevalence of CVD among this population. Early identification of individuals at increased risk for CVD can significantly enhance preventive care [49]. Diagnostic tools, such as imaging techniques, including MRI and CT scans--particularly those utilizing stroke protocols--are valuable methods for detecting cardiovascular risks and structural abnormalities in OA patients [50].

Pharmacological and Surgical Strategies for Managing Cardiovascular Risk in Osteoarthritis

Effective pharmacological management in OA extends beyond symptomatic relief to include strategies that minimize cardiovascular risks and optimize overall health outcomes [51]. While NSAIDs are a cornerstone of OA symptom management, they have been associated with an increased risk of CVD. Alternatives, such as topical NSAIDs or acetaminophen, may provide symptom relief with potentially fewer cardiovascular risks [51]. Emerging therapies, such as intra-articular injections of hyaluronic acid or corticosteroids, offer effective pain management for OA without significantly increasing the risk of CVD [51].

Beyond pain control, addressing metabolic syndrome--frequently observed in OA patients--is crucial for reducing both cardiovascular events and OA progression [16]. Pharmacological interventions, such as antihypertensives, diabetes medications, and statins, have shown benefits for both musculoskeletal and cardiovascular health [16,17]. Effective management of these metabolic abnormalities is essential, as interventions targeting these factors not only reduce the incidence of cardiovascular events but may also slow OA progression [16,17]. The use of antihypertensives, diabetes medications, and statins has demonstrated benefits in both cardiovascular and musculoskeletal health, providing secondary benefits for OA management [52]. Statins, widely used for dyslipidemia and cardiovascular protection, have been associated with a potential reduction in joint degeneration [53]. Observational studies suggest that statins may slow the progression of OA, potentially delaying the need for total joint replacement [53]. This effect is particularly relevant in elderly OA patients with concurrent ischemic heart disease or metabolic disorders, where systemic inflammation plays a key role in disease pathology [53]. Similarly, angiotensin II (Ang II) has been implicated in chondrocyte apoptosis and cartilage degradation in OA [54]. Angiotensin-converting enzyme (ACE) inhibitors, commonly prescribed for hypertension and heart failure, may exert chondroprotective effects by modulating Ang II activity [54]. This dual benefit makes ACE inhibitors a potential therapeutic option for managing both cardiovascular risk and OA progression [54].

Given the polypharmacy commonly encountered in OA patients--particularly the concurrent use of analgesics and cardiovascular medications--careful monitoring for drug interactions is essential to ensure both efficacy and safety, preventing adverse events [55].

Polmacoxib, a novel first-in-class oral NSAID, uniquely combines COX-2 and carbonic anhydrase (CA) inhibition to deliver potent analgesic and functional benefits in OA patients while minimizing traditional NSAID risks [56]. As a selective COX-2 inhibitor, it significantly lowers gastrointestinal complications common with nonselective NSAIDs [56]. Its additional CA inhibition introduces tissue-specific transport-dampening COX-2 activity in CA-rich cardiovascular and renal tissues--a mechanism thought to reduce COX-2-associated cardiovascular risks [56]. It has been demonstrated that polmacoxib 2 mg offers comparable efficacy to celecoxib with fewer GI adverse events and good long-term tolerability [56]. Additionally, polmacoxib's potent COX-2 selectivity with CA binding may make it the most tissue-targeted COX-2 inhibitor available, offering a favorable safety margin that is ideal for long-term OA management [56].

Patient education and structured follow-ups play a pivotal role in optimizing pharmacological treatment outcomes. Enhancing patient awareness of the OA-CVD relationship can improve adherence to lifestyle modifications and medication regimens while promoting early symptom reporting for timely interventions. Regular follow-ups further support comprehensive disease management, ensuring proactive adjustments to therapy as needed [57].

The study by Ravi et al. highlights that total joint arthroplasty (TJA) contributes to cardiovascular health in OA patients through multiple mechanisms [44]. Pain reduction post-TJA helps normalize autonomic function by reducing sympathetic overactivity, restores cortisol regulation via the HPA axis, and improves sleep architecture--all of which lower cardiovascular strain [44]. It also reduces reliance on NSAIDs, which are associated with increased CVD risk due to COX-2 inhibition and a prothrombotic state [44]. Data show a dose-response relationship where reduced NSAID use post-TJA correlates with fewer cardiovascular events [44]. Restoration of physical activity after TJA enhances functional mobility, promotes endothelial function, improves insulin sensitivity, and supports healthier lipid profiles [44]. For knee OA patients, the regained ability to perform weight-bearing exercises further amplifies cardiovascular benefits [44]. Finally, TJA may reduce systemic inflammation by eliminating the inflamed joint, decreasing cytokines like IL-6, TNF-α, and CRP, which are linked to endothelial dysfunction and atherosclerosis [44]. CRP reduction after periarticular steroid use during TJA and the potential for breaking chronic inflammatory cycles suggest long-term cardiovascular advantages [44].

Limitations

Despite the growing body of evidence linking OA and CVD, several limitations must be acknowledged. The heterogeneity in study designs, patient populations, and diagnostic criteria introduces variability in findings, making it challenging to establish standardized clinical guidelines. Residual confounding remains a concern, as OA frequently coexists with metabolic syndrome, obesity, and physical inactivity, all of which independently contribute to CVD risk. This overlap complicates efforts to isolate the direct impact of OA on cardiovascular outcomes. Additionally, while traditional cardiovascular risk assessments such as the Framingham Risk Score provide valuable insights, they may not fully capture OA-specific cardiovascular risks, necessitating more refined diagnostic approaches. The long-term effects of pharmacological interventions--particularly NSAIDs, statins, and ACE inhibitors--on both OA progression and cardiovascular outcomes require further investigation through large-scale, longitudinal studies. Furthermore, while surgical interventions, such as total joint arthroplasty, have demonstrated indirect benefits on cardiovascular health, their long-term impact on cardiovascular morbidity and mortality remains unclear. Future research should focus on elucidating the mechanistic pathways linking OA and CVD, improving risk stratification tools, and optimizing multidisciplinary treatment strategies.

## Conclusions

The relationship between OA and CVD is multifaceted, driven by shared risk factors, systemic inflammation, and functional decline. Given the high prevalence of CVD in OA patients, comprehensive cardiovascular risk assessment should be an integral part of OA management. Optimizing cardiovascular health in this population requires a multidisciplinary approach that encompasses targeted lifestyle modifications, personalized pharmacological strategies, and, when appropriate, surgical interventions. Emerging therapies, including selective NSAIDs with improved safety profiles and pharmacological agents with dual benefits for both OA and CVD, hold promise for enhancing patient outcomes. Future research should prioritize clarifying causative mechanisms, refining predictive models, and developing personalized treatment pathways to mitigate cardiovascular complications in OA patients. By integrating cardiovascular risk management into routine OA care, clinicians can improve both musculoskeletal function and long-term cardiovascular health, ultimately enhancing overall patient well-being.
